# Co-occurrence patterns of bacteria within microbiome of Moscow subway

**DOI:** 10.1016/j.csbj.2020.01.007

**Published:** 2020-02-01

**Authors:** Natalia S. Klimenko, Alexander V. Tyakht, Stepan V. Toshchakov, Margarita A. Shevchenko, Aleksei A. Korzhenkov, Ebrahim Afshinnekoo, Christopher E. Mason, Dmitry G. Alexeev

**Affiliations:** a Knomics LLC, Skolkovo Innovation Center, Bolshoy Bulvar Str., Building 42, Premise 1, Room 1639, Moscow 143026, Russia; b Center for Precision Genome Editing and Genetic Technologies for Biomedicine, Institute of Gene Biology, Russian Academy of Sciences, Vavilova Str., 34/5, Moscow 119334, Russia; c National Research Center “Kurchatov Institute”, Akademika Kurchatova Sq., 1, Moscow 123182, Russia; d Immanuel Kant Baltic Federal University, Universitetskaya Str., 2, Room 106, Kaliningrad 236040, Russia; e Department of Physiology and Biophysics, Weill Cornell Medicine, New York, NY, USA; f The HRH Prince Alwaleed Bin Talal Bin Abdulaziz Alsaud Institute for Computational Biomedicine, Weill Cornell Medicine, New York, NY, USA; g The WorldQuant Initiative for Quantitative Prediction, Weill Cornell Medicine, New York, NY, USA; h The Feil Family Brain and Mind Research Institute, Weill Cornell Medicine, New York, NY, USA; i ITMO University, Kronverkskiy Pr., 49, St. Petersburg 197101, Russia; j Novosibirsk State University, Pirogova Str., 1, Novosibirsk 630073, Russia; k Atlas Biomed Group, 92 Albert Embankment, Lambeth, London SE1 7TT, UK

**Keywords:** Urban microbiome, Subway, Built environments, Co-occurrence patterns, 16S rRNA, Biosurveillance

## Abstract

Microbial ecosystems of the built environments have become key mediators of health as people worldwide tend to spend large amount of time indoors. Underexposure to microbes at an early age is linked to increased risks of allergic and autoimmune diseases. Transportation systems are of particular interest, as they are globally the largest space for interactions between city-dwellers.

Here we performed the first pilot study of the Moscow subway microbiome by analyzing swabs collected from 5 types of surfaces at 4 stations using high-throughput 16S rRNA gene sequencing. The study was conducted as a part of The Metagenomics and Metadesign of the Subways and Urban Biomes (MetaSUB) project.

The most abundant microbial taxa comprising the subway microbiome originated from soil and human skin. Microbiome diversity was positively correlated with passenger traffic. No substantial evidence of major human pathogens presence was found. Co-occurrence analysis revealed clusters of microbial genera including combinations of microbes likely originating from different niches. The clusters as well as the most abundant microbes were similar to ones obtained for the published data on New-York City subway microbiome.

Our results suggest that people are the main source and driving force of diversity in subway-associated microbiome. The data form a basis for a wider survey of Moscow subway microbiome to explore its longitudinal dynamics by analyzing an extended set of sample types and stations. Complementation of methods with viability testing, “shotgun” metagenomics, sequencing of bacterial isolates and culturomics will provide insights for public health, biosafety, microbial ecology and urban design.

## Highlights

• Central components of subway samples are soil and human skin microbes.
• Microbial diversity is correlated with passenger traffic.
• Co-occurring groups of microbes resonate with other studies of subway microbiome.
• No substantial evidence of major pathogens presence was detected.

## 1 Introduction

Microbial communities are major determinants of human organism homeostasis. The most important of them – host-associated microbiome – are initially seeded by vertical transmission from mother and establish after a series of successions during infancy and puberty [17]. From the outside, it is being constantly modulated by dietary habits on short and long-term scales [27] and replenished by members of food microbial consortia [51]. Humans are also exposed to diverse environmental microbiota – however, the exposure is significantly reduced for urban inhabitants who comprise more than half of the world population [41].

Metropolitan dwellers constantly interact with each other and their surroundings, thus contributing to the unique complex microbial community structures of the urban environment. During these interactions, humans serve both as sources of their indigenous microbes and as carriers of external microbes, while serving as potential targets of opportunistic species. Previous studies show that the microbiome of indoor environment not only contains unique signature of their dwellers but also preserves information about the ways of interaction [32]. Examples include prediction of the body part a surface most frequently contacts with [21] or building occupancy [24] from community composition.

As modern humans spend more and more of their time indoors, the importance of the interactions between indoor microbiome and human health is becoming more relevant. Higher rates of allergic asthma, diabetes and atopy in the Western world are suggested to be linked to insufficient exposure to environmental microbes, especially at a younger age [15], [35], [50]. Certain microbes can synthesize volatile organic compounds that can cause allergic reactions [9], [13], [44]. Widespread use of antimicrobial compounds in the cities with high concentration of population contributes – in part, via human microbiota [54] and in built environments [34] – to the selection of multiple-drug resistant pathogens that represent a global health hazard. These risks can be attenuated by adequate hygiene practices, physical barriers and maintenance of microbial diversity of the communities to which humans are exposed. However, there is very limited knowledge about the impact of specific environmental bacteria on our health, and it is still difficult to determine the constituents of a “reference” environmental community that might confer health benefits [23].

Among the multitude of urban environments, transportation systems represent a uniquely centralized place. Their intense use leads to a high number of dense interactions between inhabitants across the center and periphery of cities, forming a vast microbial dissemination network. Thus, surveys of microbial communities associated with transportation systems are of particular interest for healthcare and security, as well as fundamental research. The Metagenomics and Metadesign of the Subways and Urban Biomes (MetaSUB) project was established in 2015; one of its goals is to design an integrated metagenomic map of urban environments of the cities of the world (with an emphasis on transportation systems) [37]. Global collection of information on microbial communities of transportation systems contributes to the development of novel health and ecological surveillance strategies in the cities.

Previous studies of subway microbiomes from various geographic locations highlighted specific features like diurnal variation [16], differences between communities occupying niches composed of diverse materials [25], potential for spread of pathogens and reflection of history of the examined subway locations in microbiota composition [1]. In our study, we performed a pilot analysis of the microbiome of Moscow subway (Russia) using high-throughput sequencing for the first time. The Moscow subway is ranked sixth in the world by usage, with approximately 7 million people travelling in it daily (http://www.metro-msk.ru/statisticheskie-dannye.htm).

## 2 Results

In total, 40 samples were collected according to the MetaSUB protocol by swabbing various surfaces at 4 stations of the Moscow subway system (see Supplementary Fig. 1). At each of the stations, 5 types of surfaces were sampled: floor, railings near escalator, information stand, bench and wall at shoulder level (see Supplementary Table 1).

All 40 sequenced samples received sufficient coverage (>3000 reads after filtering using DADA2 algorithm [6]; rarefied to this number of classified reads). Negative controls contained negligible amount of reads (4 in DNA extraction – and 6 in PCR negative controls, respectively) suggesting that contamination did not occur at the respective experimental stages.

Basic analysis (quality control and composition profiling) and factor analysis (statistical and visual comparison of microbiome composition with the meta-data) were performed using Knomics-Biota platform [14]; the respective project with interactive reports is publicly available online at https://biota.knomics.ru/moscow-subway-pilot (project ID 1031).

**Fig. 1:**
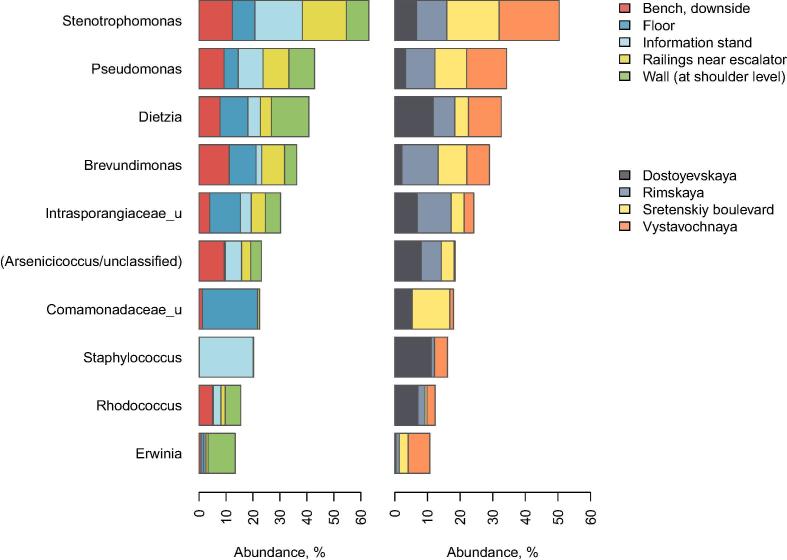
Top ten most prevalent microbial genera for different subway stations and sample types. Barplot height corresponds to the average genus abundance for all samples from a specific group. In the left and right columns of barplots, colors represent the types of surfaces and stations, respectively (see legend on the right). The “NNN_u” notation refers to the “unclassified genus(-era) from family NNN”.

All of the identified microbes belonged to *Bacteria* kingdom (no *Archaea* were detected). The major genera are shown in Fig. 1.

### 2.1 Universally prevalent subway microbes

Some microbial taxa were quite uniformly distributed among different types of surfaces while others were niche-specific. The most prevalent genera – detected in most niches and stations – included *Dietzia, Brevundimonas, Pseudomonas, Arsenicicoccus, Stenotrophomonas* and unclassified genera from *Intrasporangiaceae* family. These aerobic taxa are widespread in the environment, particularly, soil and ocean. The *Dietzia* genus can also be a human skin commensal [52]. *Pseudomonas*, *Brevundimonas* and *Stenotrophomonas* have been observed among the most prevalent bacteria found in the New York City subway study [1].

As 16S rRNA gene sequencing does not always allow to classify microorganisms at the level of species, we used TaxMan service [4] to improve the precision of identification beyond one provided by mapping to a complete 16S rRNA gene sequence OTU database. As a result, it was found that *Brevundimonas* genus was represented solely by *Brevundimonas diminuta, Pseudomonas* – in part by *P. stutzeri* and *P. veronii*, and *Stenotrophomonas* – in part by *S. acidaminiphila* species. Some of these bacteria possess specific metabolic capabilities. For instance, *P. stutzeri* manifests a broad phenotypic and genotypic diversity and can participate in degradation of aromatic compounds, in particular, environmental pollutants [20], [31].

Subway surfaces are considered a possible channel for transmission of pathogens between people via direct or indirect hand contact. For preliminary assessment of the pathogen carriage potential of the Moscow subway samples, we compiled a list of around 60 pathogenic taxa from two representative online resources – HPSC (Ireland) and NIH NIAID (US) (“Health Protection Surveillance Centre” https://www.hpsc.ie/, “NIH: National Institute of Allergy and Infectious Diseases | Leading Research to Understand, Treat, and Prevent Infectious, Immunologic, and Allergic Diseases” http://www.niaid.nih.gov.) Basing on this set, a list of pathogenic species reliably detectable using the selected format of sequencing (i.e. the taxa for which the amplified region of 16S rRNA gene was not found to be identical to any other non-pathogenic taxon) was obtained using TaxMan software [4]. Overall, 10 taxa could be in this sense precisely identified by our method (see Supplementary Table 2). The analysis of sequences showed that none of these taxa were detected in the collected samples, except for noise-level detection of *Clostridium perfringens* in the only sample (Dostoyevskaya station, railings near escalator surface; 0.1%; the exact 253 bp long sequence is given in Supplementary Table 3). *C. perfringens* is an obligate anaerobe, but can form spores to survive in aerobic conditions. Despite the fact that the species is referred to as the most widely distributed pathogenic microbe in nature [33]), it is also considered a part of normal gut flora of humans and animals. Its highly varying virulence potential encoded in genome [45], [46] cannot be reliably assessed using 16S rRNA sequencing.

### 2.2 Microbiome richness depends on surface type and station

**Fig. 2:**
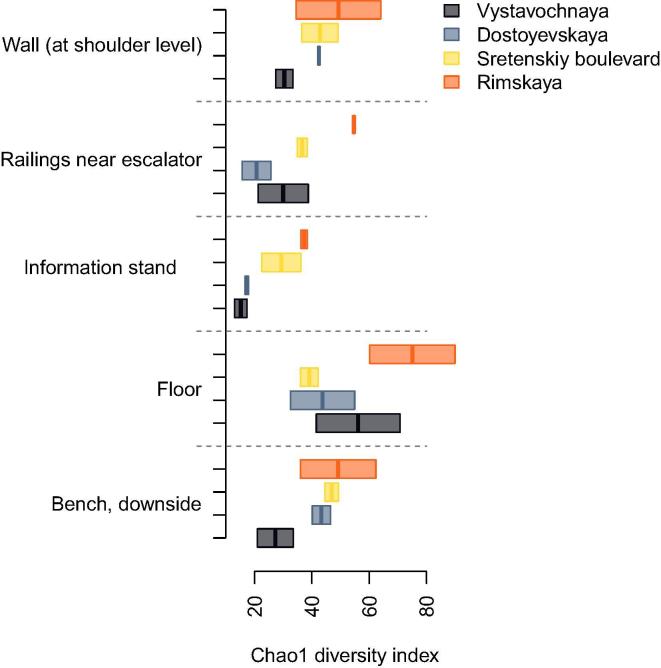
Variation of alpha-diversity (Chao1 diversity index) across stations and surface types.

**Fig. 3:**
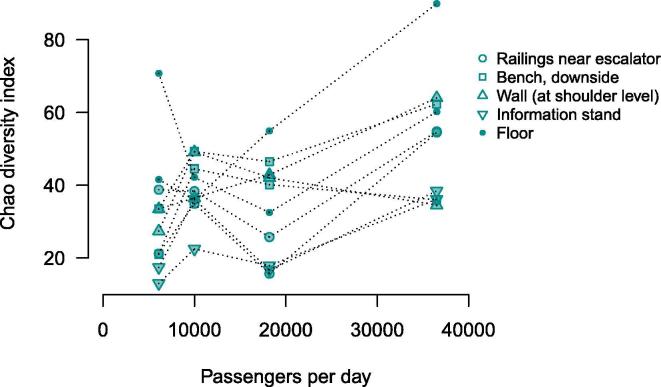
Association between daily passenger traffic and alpha-diversity. The four stacks of samples correspond to the stations in the following order: Vystavochnaya, Sretenskiy boulevard, Dostoyevskaya and Rimskaya.

We analyzed how richness of microbiome (alpha-diversity) varies across the stations and surface types. Among the stations, a significant association was observed only for Rimskaya station: it was found to manifest the highest overall alpha-diversity (Chao1 index, Mann-Whitney test, FDR-adjusted p = 0.03, N = 40, Fig. 2). The finding was replicated with two other alpha-diversity metrics (Shannon diversity index – FDR-adjusted p = 0.0013, Faith's phylogenetic diversity – FDR-adjusted p = 0.05, Supplementary Fig. 2). Interestingly, this station has the highest passenger traffic per day (avg. 36,500 people daily vs. 6500–18200 at the others) (http://www.metro-msk.ru). There was also a positive correlation between number of passengers per day and alpha-diversity itself (Chao1 index, Spearman correlation coefficient r = 0.39, FDR-adjusted p.value = 0.01, n = 40) (Fig. 3). The result was replicated with other alpha-diversity metrics (Shannon diversity index – p = 0.0014, Faith's phylogenetic diversity index p = 0.05). Similar associations with passenger transit were observed in previous studies of transport systems [1]. No significant associations of alpha-diversity were detected neither with the depth nor with the opening date of the stations.

Analysis of associations between surface types and alpha-diversity revealed that the samples collected from the floor are characterised by higher diversity compared to other samples (Mann-Whitney test, FDR-adjusted p = 0.07, N = 40, Fig. 2). At the same time, samples collected from the information stands had lower diversity values (FDR-adjusted p = 0.03, N = 40, Fig. 2). This association was still significant using Faith's phylogenetic diversity metric (FDR-adjusted p < 0.05, Supplementary Fig. 2) but not Shannon diversity index (FDR-adjusted p > 0.1, Supplementary Fig. 2).

### 2.3 Community composition depends mainly on surface type

**Fig. 4:**
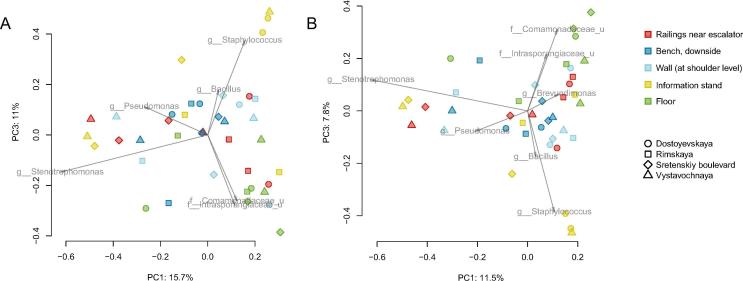
Distribution of microbial community structures across Moscow subway stations and sample types using two metrics (principal coordinates analysis, PCoA): Bray-Curtis metric (A) and Jaccard metric (B). Arrows in the bi-plots show major microbial drivers of the observed variance.

**Fig. 5:**
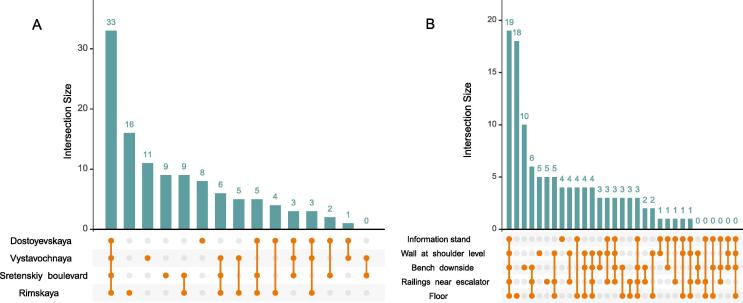
UpSet plots illustrating quantitative intersection of the sets of microbial genera across the stations (A) and surfaces (B). The numbers above the bars show the number of common genera between the groups of samples marked below the bars.

We examined the associations of microbiome composition with various factors using PERMANOVA and two beta-diversity metrics – Jaccard index and Bray-Curtis distance – to assess qualitative and quantitative effects, respectively (Fig. 4). For both metrics, the most contributing factor was the surface type (R^2^ = 14% for Bray-Curtis metric and 13% for Jaccard metric, N = 40 samples). The floor surface samples contained the highest number of unique genera – not detected on other surfaces (Fig. 5). Among the stations, the largest number of unique genera was for Rimskaya station (as mentioned above, this station had the highest passenger traffic).

Differences in relative abundance of individual taxa between stations/surfaces are listed in Supplementary Table 3 (MaAsLin method, FDR adjusted p < 0.05). The subway floors were significantly different from the other surfaces by levels of *Actinomycetaceae*, *Rhodobacteraceae*, *Enterobacteriaceae, Comamonadaceae*, *Clostridiaceae* families and *Gammaproteobacteria* phylum. The information stands had the highest levels of *Bacillus* genus. Finally, the passenger traffic was associated with the abundance of soil bacteria from *Microbacteriaceae* and *Nocardiaceae* families.

### 2.4 Patterns of co-occurrence suggests mixing of microbes from different niches

**Fig. 6:**
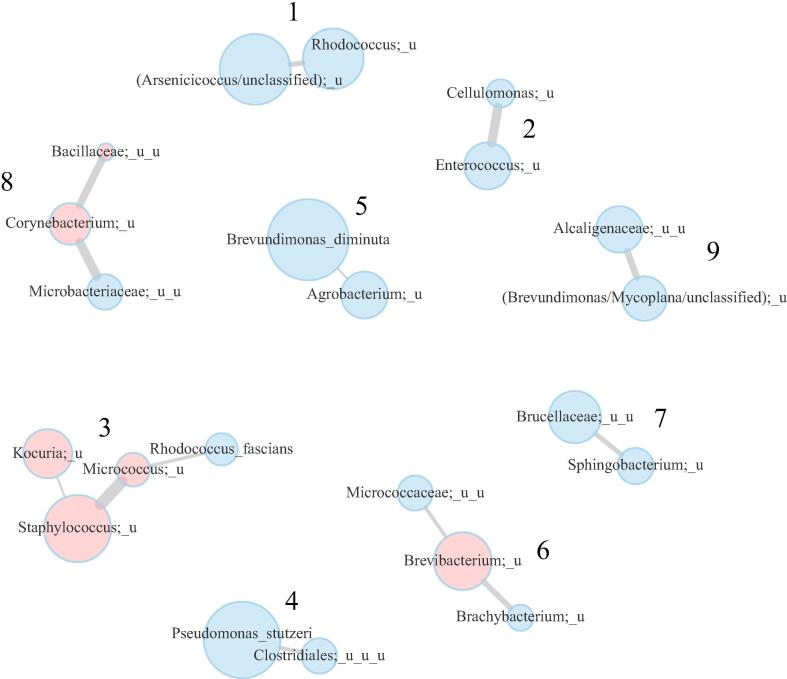
Microbial clusters (co-occurrence groups of species) calculated using SPIEC-EASI. Vertex diameter is proportional to the abundance of the taxon. The taxa likely originating from human microbiome are shown in pink. (For interpretation of the references to colour in this figure legend, the reader is referred to the web version of this article.)

In order to reduce the dimensionality of analysis and explore the potential of ecological interactions between the microbes in subway microbiome, we analyzed co-occurrence of microbial species according to the respective relative abundance values. Overall, 9 clusters of co-occurring bacteria were detected across all samples containing 4 ± 2 species (Fig. 6).

The majority of the discovered clusters include various ensembles of soil bacteria: these are the first, second and fourth co-occurrence clusters.

Cluster #1 members (*Arsenicicoccus/unclassified*, *Rhodococcus*) are distinguished by interesting metabolic capacities: *Arsenicicoccus* was recovered from an arsenic-enriched environment [10], [43], some species of *Rhodococcus* are also arsenic-resistant [5], [42] and can metabolize harmful environmental pollutants [52].

In the cluster #2, there are unclassified species from *Enterococcus* and *Cellulomonas* genera. Although *Enterococcus* is primary associated with human and animal gut microbiota and is frequently used as fecal indicator bacterium, it was also shown to be widespread in other environments such as soils [39]. As for *Cellulomonas* species, their major habitats are generally considered to be soils, as well as decayed wood, cellulose-containing material and municipal waste [52]*.*

Interestingly, cluster #4 include obligate anaerobic *Clostridiales*. Other member is *Pseudomonas stutzeri* which can participate in degradation of aromatic compounds and grow under both aerobic and anaerobic conditions. Detection of strict anaerobes in the subway in the presence of O_2_ might be due to the fact that our method detects both viable and dead bacteria, or because they are present as spores (as some *Clostridiales* are capable of spore forming.)

Clusters #5, #7 and #9 include bacteria that are all widespread in the environment. Their possible sources include soil, water, plant surfaces, animal and human organisms [52].

Clusters #3, #6 and #8 presumably include some host-associated bacteria. Among the members of the cluster #3, there are typical dwellers of human oral cavity and skin such as unclassified species from *Kocuria, Micrococcus* and *Staphylococcus* [28], [30], [47]. At the same time, there is at least one typical soil-borne bacteria in this co-occurrence cluster – *Rhodococcus fascians* (some strains of which are phytopathogens.)

Most of cluster #6 bacteria (*Micrococcaceae*, *Brachybacterium*) are associated with highly diverse range of ecological niches ranging from water and soil to animals and built environments. However, habitat of species from *Brevibacterium* genus is not so diverse, being characterized by a relatively high salt levels (marine water), food- (dairy products) or human-associated (skin); apparently, human skin is the most likely source of this bacterium in the subway [52].

Cluster #8 includes *Corynebacterium* known to be one of the major members of human skin microbiome. The cluster also includes a soil-borne *Microbacterium* genus. Interestingly, cross-feeding with other skin microbes has been shown for *Corynebacterium* as it lacks the ability to utilize macromolecular compounds [30].

To investigate if there are global co-occurrence patterns typical for subways, we applied this analysis to the dataset from the New York City (NYC) subway microbiome study [1] and compared the results with the patterns we identified in Moscow subway (Supplementary Fig. 3). Although the NYC subway study employed a different microbiome profiling method (“shotgun” metagenomics), some parallels were still observed. The majority of co-occurring bacterial clusters from the NYC subway study included phylogenetically related bacteria, but the largest cluster (including 10 species) was very similar to cluster #3 from the Moscow subway. Each of the clusters included species from *Kocuria*, *Micrococcus* and *Staphylococcus* genera. As “shotgun” metagenomics allow a more accurate taxonomic profiling than 16S rRNA sequencing, it was possible to identify the respective NYC cluster drivers down to the species level as *Kocuria rhizophila*, *Micrococcus luteus* and *Staphylococcus hominis* and *saprophyticus*.

In the Moscow subway, the relative abundance of co-occurring bacteria clusters (sum of the levels of their member taxa) was not associated neither with the station nor with the surface type (MaAsLin, FDR adjusted p > 0.1). There were some significant associations between clusters and alpha-diversity. Clusters #5 (*Brevundimonas diminuta* and *Agrobacterium*), #4 (*Pseudomonas stutzeri* and unclassified *Clostridiales*) and #9 (unclassified *Alcaligenaceae* and unclassified *(Brevundimonas/Mycoplana)*) were positively associated with alpha-diversity, while cluster #3 (unclassified *Kocuria, Micrococcus, Staphylococcus* and *Rhodococcus fascians*) was associated negatively (Spearman correlation test, FDR-adjusted p < 0.05). Interestingly, the negatively associated cluster included microbes typical for human skin (*Micrococcus*, *Kocuria*), while the positively-associated ones contain soil-borne bacteria (*Pseudomonas stutzeri, Agrobacterium*).

## 3 Discussion

This study is the first pilot survey of the Moscow transit system microbiome. Although the number of samples is limited and they were collected at a single time point, it already shows certain interesting patterns worth further investigation, like the impact of passenger traffic and surface type as well as stable co-occurrence patterns between the microbes. The most prevalent microbes of Moscow subway surfaces are typical soil dwellers and skin-associated microorganisms. These results are quite expected because the surfaces are subject to regular cleaning and most of the biological material is introduced to them from the outside on passengers’ shoes and hands. Many of these bacteria were previously identified as major members of microbiota in other subways of the world – for example, *Pseudomonas*, *Brevundimonas* and *Stenotrophomonas* in New York [1]. Interestingly, independently of climate, geography and many other factors, these specific clades of microbes appear to be universally adapted to unusual subway conditions: lack of light and small amount of carbon sources, constant introduction of novel microbes from outside. Our results extend the volume of the datasets that can be subject to meta-analysis of subway microbiota from many cities to come up with mechanisms that allow these microbes to appear and potentially survive in such environment. Most high-abundant microorganisms we detected are aerobic heterotrophs. Some can degrade organic industrial pollutants (*Rhodococcus*, *Pseudomonas stutzeri* [31], [52] and survive in polluted environments (*Arsenicicoccus* [43]. Their prevalence and abundance might be potential markers and indicators of dynamic changes in urban ecology. Moreover, it has been shown that atmospheric pollutants can be accumulated in biofilms and serve as nutrients for primary colonizing chemoorganotrophic bacteria which then produce other organic compounds for further colonization [19].

Application of a microbial co-occurrence network approach revealed existence of several connected components – clusters of co-occurring microbial genera. Some of the clusters included bacteria likely coming from different source types – for example, those associated with human skin and oral cavity (*Staphylococcus, Micrococcus, Kocuria*) together with soil microbes (*Rhodococcus*) in cluster #3. Interestingly, a cluster very similar to cluster #3 was also identified during similar analysis in the New-York subway dataset [1]. Moreover, *Staphylococcus hominis, Micrococcus luteus* and *Kocuria rhizophila* were previously shown to form a co-occurence cluster in the study of human skin microbiota [8]. *Kocuria* and *Staphylococcus* had been previously characterized together as the most abundant bacteria on the floors and mats of the fitness centers environment [53]. Other examples of clusters containing presumably bacteria from various sources are eighth and sixth clusters. The eighth cluster includes an interesting association between *Corynebacterium* and unclassified *Bacillaceae*. Previously it was shown that *Corynebacterium* lacks the ability to utilize macromolecular compounds present on the skin, but rather participates in cross-feeding relationship with bacteria that can metabolize them with high efficiency, such as *Staphylococcus* [30]. In our data, we found an association between *Corynebacterium* and unclassified *Bacillaceae*, a clade taxonomically close to *Staphylococcus* – suggesting a potential for symbiotic interactions.

Some of the clusters were found in more diverse communities (clusters #4 and #5), while others - in less diverse ones (#3). The clusters associated with richer community contained soil-borne microbes. The only cluster significantly higher represented in the communities with lower diversity was the cluster #3. As mentioned above, this cluster contains microbes associated with human skin and oral cavity.

It is tempting to interpret the identified co-occurrence clusters of microbial taxa as potential cooperatives – guilds of taxa that interact with each other – to speculate how these microbes can adapt to such unusual environment as subway. The observed co-occurrence patterns between the microbes from different niches could also suggest onset of novel symbiotic interactions. However, recent analyses show that one should approach such covariance-based results with caution. Experiments on realistic simulations showed that interaction patterns are inadequately predicted using co-occurrence networks [22]. The other challenges include complex features of microbiome sequencing data (like compositionality and zero-inflation), spurious effects of data transformations, nonlinear dynamics of microbial taxa, indirect associations, frequent asymmetry of ecological interactions, latent factors driving the dynamics, as well as batch effects. It follows that the co-occurence patterns can be useful for reducing the dimensionality of the data and the number of potential hypotheses to be tested, but it is also important to take into account mechanistic constraints and temporal dynamics and the ultimate validation of ecological interactions between the microbes should be performed experimentally [7].

Previous studies of subway microbiota did not reveal any significant presence of microorganisms with definitive pathogenic potential reflecting its relative biosafety. We observed similar results for Moscow subway, as far as the sample size and precision of the applied method allowed. The analysis was quite encompassing, with the list being based on two authoritative sources. Although the pathogen list could be extended by adding selected taxa from other databases, the method definitely does not provide complete scope, as many pathogens – for example, *Escherichia coli* – can carry identical 16S rRNA genes but at the same their role can vary from probiotic through commensal to pathogen [49].

Investigations of the microbial populations, along with studies of their metabolites, can help to understand the microbial ecology of microbiome in large cities and reveal signatures of community adaptation to new environmental conditions. Indeed, since previous studies have shown that microbes can survive in built environments [1] and be transferred to humans [23], these results help frame and expand our understanding of this important and unique environment.

Although our study contains a relatively small number of samples, the findings support the feasibility and value of future larger surveys in subway systems of Moscow and other cities in Russia and worldwide. Such urban microbiome sequencing data can be easily subject to exploratory comparative analysis in interactive systems like Knomics-Biota – especially when augmented with meta-data in a unified format like proposed by the MetaSUB initiative.

There are a few inherent limitations of 16S rRNA surveys. Firstly, such format of sequencing provides information only on prokaryotic (bacterial and archaeal) abundance in the community, while information on protozoa and fungi would also be interesting in the context of subway microbiome. Other primers targeting fungal ITS (internally transcribed sequence) or 18S rRNA sequence can help to address these issues.

Secondly, there is a lack of information about the gene content of the microbial species detected in the community structure. Such information would be important for assessing more realistically the antibiotic resistance and virulence potential of the urban microbiome. It can be achieved by applying “shotgun” metagenomics. The method would also allow to detect the above-mentioned eukaryotes as well as viruses – the former being of high interest in the context of public health.

Thirdly, the analysis does not allow neither to distinguish viable and dead microbial cells nor to assess the total microbial load – thus limiting the conclusions about the ecological interactions between them as well as their potential effect on humans. To overcome these issues, sequencing-based viability assays with reagents like PMA (propidium monoazide) and qPCR analysis of total microbial abundance on standardized samples can be applied, respectively.

Overall, out pilot initiative presents a unique dataset that contributes to catalogizing the composition and elucidating the ecology of microbiome of built environments, in particular the transportation systems. It offers a basis for further advanced survey of Moscow subway microbiome in collaboration with public health, hygiene and biosurveillance experts and using a richer repertoire of methods like culturomics, viability testing, quantification of cells, whole-genome sequencing and drug resistance/virulence screening – as well as by assessing longitudinal dynamics of multiple sample types from surfaces and air.

## 4 Conclusions

Our pilot analysis of microbiome associated with Moscow subway using surface swabs showed that their taxonomic composition has many features common with ones described for the transit systems of other cities. There is an enrichment of soil-dwelling and skin-associated taxa – including those that are able to degrade environmental pollutants – as well as many plant-associated pathogens and symbionts likely brought from the outside with soil. Together with the observed correlation between alpha-diversity and passenger traffic, these observations provide further evidence that the indoor microbial ecosystem is a dynamic structure reflecting the changing impact of external sources of microbes.

The clusters of microbial taxa identified during co-occurrence network analysis, particularly, those combining genera from different environments, represent a priority list of potential microbial ecological interactions to be investigated basing on more advanced approaches including experimental validation.

Finally, our results extend the global map of microbial communities associated with urban environments, contribute to the understanding of the factors affecting their composition as well as to improving healthcare, biosafety and critical infrastructure security.

## 5 Methods

### 5.1 Sample collection

The samples were collected on Friday, June 10, 2016 in a single session between 1:30 pm and 3:30 pm (local time). The collection time was after the end of the morning rush hour. There was no precipitation on this day, the outdoor ambient temperature was about 20 °C, the relative humidity was 30%. Four stations of the Moscow subway were sampled: Vystavochnaya, Dostoevskaya, Sretenskiy boulevard and Rimskaya. All stations are located underground and have at least one direct exit to the street. For all stations, data on the number of passengers per day, depth and opening date were collected.

At each of the stations, 5 types of surfaces were sampled: floor, railings near escalator, information stand, bench and wall (at shoulder level). For each type of surface, two samples were collected from different places at the same station totally resulting in 40 samples. For each sample surface, material type was also recorded. Collection was performed using COPAN ESwab kits (Copan Diagnostics, Murrieta, CA, USA) according to the guidelines of MetaSUB consortium (http://metasub.org/). The samples were sent to the laboratory within 48 h of collection where they were frozen. DNA extraction and sequencing procedure is described in Supplementary Methods. The rRNA-complementary parts of the primers were standard F515-R806 sequences with slight modifications aimed to improve the coverage of environmental taxa [26], [36]. Additionally, two negative controls (NC) were prepared and sequenced – one DNA extraction negative control and one PCR negative control. Ultimately, the NC sample sequences contained 4 reads suggesting lack of contamination.

### 5.2 Data analysis

The obtained reads were analyzed using Knomics-Biota platform (https://biota.knomics.ru/) [14], the exact sequence features were obtained using DADA2 filtering algorithm [6]. The database for taxonomic assignment was prepared by applying TaxMan service [4] to GreenGenes v13.5 [12] database (using F515-R806 primers) with further clustering 97% identical sequences using CD-HIT software version 4.8.1 [18]. When two or more taxa from GreenGenes database could not be resolved with the used primers, a slash (“/”) character was used to denote such ambiguity. Taxonomic composition was obtained by applying Naive Bayes classifier implemented in QIIME 2 trained on the preprocessed database [3]. All readsets were randomly rarefied to 3000 reads per sample. Alpha-diversity was assessed via Chao1, Shannon and Faith's phylogenetic diversity metrics using QIIME 2. Besides standard analytical reports generated in Knomics-Biota reports, additional data analysis was performed as described below.

All statistical analysis was performed in R programming language, version 3.6.0 [48]. Associations of alpha-diversity with metadata factors was assessed using Mann-Whitney test. For each factor, the samples with specific value were compared with samples having all other values (for example, samples from Rimskaya station against samples from all other stations). Multiple comparison adjustment was performed using Benjamini-Hochberg method. Association between alpha-diversity and number of passengers per day was tested by calculation of Spearman correlation coefficient with further testing for statistical significance using AS algorithm [2].

Beta-diversity (pairwise dissimilarity between the community structures) was estimated using Bray-Curtis dissimilarity and Jaccard index. To test if any of the factors impact community structure generally, permutational multivariate analysis of variance (PERMANOVA) analysis was applied [40] for each of the two metrics. Links between individual taxa and factors were checked using MaAsLin package version 0.0.4 [38] with adjustment for the other factors (effect of sample type was evaluated with adjustment for the station, and vice versa). The analysis was performed separately for each of the factors with default settings of the algorithm (including filtering, outliers removal and arcsin-sqrt transform of relative abundance taxa) with disabled boosting step. Intersections of sets of genera detected at surfaces and stations were visualized using UpsetR package version 1.4.0 [11].

Identification of co-occurring bacterial groups was performed using SPIEC-EASI package version 1.0.6 [29] at the species level. Species having <10 reads per sample on average or having non-zero abundance in <20 percent of samples (equivalent to 8 samples) were excluded. For the similar analysis of the NYC subway data, the threshold was set lower (1% equivalent to 20 samples, respectively) due to different total number of samples for achieving the level of graph granularity comparable to Moscow graph. Meinshausen-Bühlmann’s selection method (mb) was used for neighbours identification and StARS algorithm (huge R package version 1.3.2 [55] – for model selection (number of subsamples = 50, number of lambda iterations = 10, minimum lambda ratio = 0.2). Clusters was determined as connected components of co-occurrence graph. Associations between clusters abundance and metadata factors were assessed using MaAsLin package version 0.0.4 [25]. Associations between clusters and alpha-diversity were assessed by calculating Spearman correlation followed by test for statistical significance [2]. Similar analysis for NYC subway data was performed using the taxa relative abundance tables obtained from the original paper.

## 6 Declarations

### 6.1 Availability of data and materials

Raw sequencing reads are deposited in the Sequence Read Archive (project ID: PRJNA495018). Repository, R scripts for data analysis and the datasets supporting the conclusions of the article are available in GitHub (https://bitbucket.org/natasha_klmnk/subway_art/).

### 6.2 Authors' contributions

D.G.A and A.V.T designed and supervised the study. S.V.T., M.A.S. and A.A.K. performed the experimental work. N.S.K and A.V.T analyzed the sequencing data. N.S.K and A.V.T performed statistical analysis. N.S.K, A.V.T. and C.E.M. wrote the manuscript. E.A. revised the manuscript. All authors approved and contributed to the preparation of the manuscript.

## Funding

The research was funded by Knomics LLC. Additionally, MetaSUB consortium provided the DNA extraction kits. The work of D.G.A. was supported by Russian Ministry of Science and Education under 5–100 Excellence Programme. N.S.K. and A.V.T. were supported by a grant 075-15-2019-1661 from the Ministry of Science and Higher Education of the Russian Federation allocated to the Center for Precision Genome Editing and Genetic Technologies for Biomedicine under Federal Research Programme for Genetic Technologies Development for 2019–2027. S.V.T. and A.A.K. were supported by a grant from Ministry of Science and Higher Education of Russian Federation allocated to the Kurchatov Center for Genome Research (grant 075-15-2019-1659).

## Declaration of interest

C.E.M. is a cofounder and board member for Biotia and Onegevity Health.

## Appendix A Supplementary data

Supplementary data 1 Supplementary methods and figures: Supplementary Figure 1 – Location of the sampled stations on the map of the Moscow subway. Supplementary Figure 2 – A, B – Variation of alpha-diversity across stations and surface types: A – Shannon diversity index and B – Faith's phylogenetic diversity. C, D – Association between daily passenger throughput and alpha-diversity: C – Shannon diversity index and D – Faith's phylogenetic diversity. Supplementary Figure 3 – Microbial clusters (co-occurrence groups of species) obtained from the data from New-York subway study.

Supplementary data 2 Supplementary Tables: Supplementary Table 1 – Sample information. Supplementary Table 2 – List of pathogens detectable with the used method. Supplementary Table 3 – Associations between microbial taxa and factors (n = 40 samples)
