# Supplementary material for: Co-occurrence patterns of bacteria within microbiome of Moscow subway
Source: Comput Struct Biotechnol J. 2020 Feb 1;18:314–22. doi: 10.1016/j.csbj.2020.01.007 (PMC7016200; doi:10.1016/j.csbj.2020.01.007)
Supplement: Supplementary data 1 — Supplementary methods and figures: Supplementary Figure 1 – Location of the sampled stations on the map of the Moscow subway. Supplementary Figure 2 – A, B – Variation of alpha-diversity across stations and surface types: A – Shannon diversity index and B – Faith's phylogenetic diversity. C, D – Association between daily passenger throughput and alpha-diversity: C – Shannon diversity index and D – Faith's phylogenetic diversity. Supplementary Figure 3 – Microbial clusters (co-occurrence groups of species) obtained from the data from New-York subway study. [file mmc1.docx]

## **Supplementary methods**

### DNA extraction and sequencing

DNA extraction from swabs was performed with ZymoBIOMICS™ DNA Mini Kit (ZymoResearch, Irvine, CA, USA) using manufacturer’s instructions. DNA samples were stored at -20°C.

For the preparation of Illumina-compatible libraries of V4 region of 16S rRNA gene, a dual-indexing primer system was used [(Fadrosh et al. 2014)](https://paperpile.com/c/Os3Gnk/6Bag). The rRNA-complementary parts of the primers were standard F515-R806 sequences with slight modifications aimed to improve the coverage of environmental taxa: forward GTGBCAGCMGCCGCGGTAA, reverse GACTACNVGGGTMTCTAATCC.

Amplification of each sample was performed in two replicates in 50 μ volumes by qPCRmix-HS™ SYBR mastermix (Evrogen, Russia). For the amplification, the extracted DNA was diluted 100 times and 1 μ of dilution was used for each reaction. To monitor the amplification in real time, PCR was performed using CFX96 real-time amplification detection system (BioRad, Hercules, CA, USA). Cycling conditions were as follows: denaturation - 98°C, 15 secs; primer annealing - 58°C, 15 secs; extension - 72°C, 25 secs. For the purpose of amplicon integrity, no melting curve analysis was performed at the final stage.

Purification of the PCR products was performed using the Cleanup Mini kit (Evrogen, Russia) for the extraction of DNA for reagent mixtures. The concentration of obtained 16S rRNA libraries in the solution was measured with Qubit® fluorometer (Thermo Fisher Scientific, Waltham, MA, USA) using Quant-iT™ dsDNA High-Sensitivity Assay Kit. The quality of the final libraries was assessed using the electrophoresis in agarose gel.

Libraries were sequenced with MiSeq™ Personal Sequencing System platform of (Illumina Inc., San Diego, CA, USA) using paired-end 250 bp reads. Demultiplexing was performed as described previously [Fadrosh et al., 2014]. After demultiplexing, all reads were subjected to stringent quality filtering, and parts of reads corresponding to 16S rRNA primers were removed using CLC Genomics Workbench 10.0 (Qiagen, Germany). After the quality trimming, the concatenation of the paired reads was performed using SeqPrep software (<https://github.com/jstjohn/SeqPrep>).

**References**

[Afshinnekoo, Ebrahim, Cem Meydan, Shanin Chowdhury, Dyala Jaroudi, Collin Boyer, Nick Bernstein, Julia M. Maritz, et al. 2015. “Geospatial Resolution of Human and Bacterial Diversity with City-Scale Metagenomics.” *Cell Systems* 1 (1): 97–97.e3.](http://paperpile.com/b/Os3Gnk/oS5b)

[Fadrosh, Douglas W., Bing Ma, Pawel Gajer, Naomi Sengamalay, Sandra Ott, Rebecca M. Brotman, and Jacques Ravel. 2014. “An Improved Dual-Indexing Approach for Multiplexed 16S rRNA Gene Sequencing on the Illumina MiSeq Platform.” *Microbiome* 2 (1): 6.](http://paperpile.com/b/Os3Gnk/6Bag)

## **Supplementary figures**


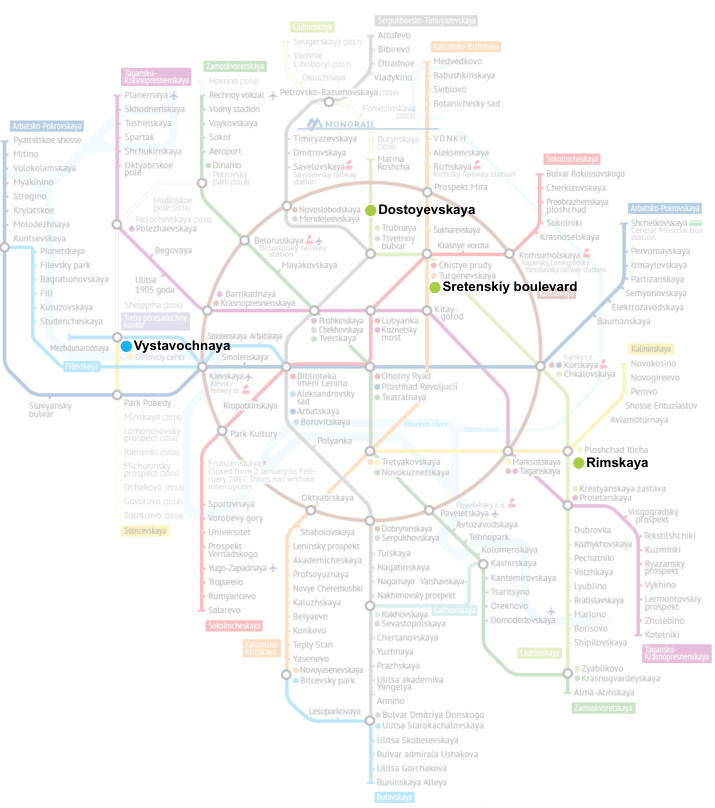


Supplementary Figure 1 - Location of the sampled stations on the map of Moscow subway.


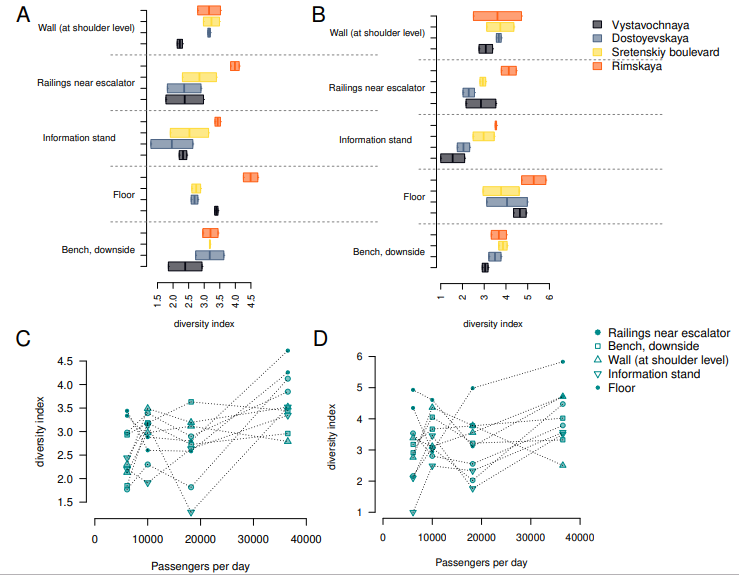


Supplementary Figure 2 - A, B - Variation of alpha-diversity across stations and surface types: A - Shannon diversity index and B - Faith's phylogenetic diversity. C, D - Association between daily passenger throughput and alpha-diversity: C - Shannon diversity index and D - Faith's phylogenetic diversity.


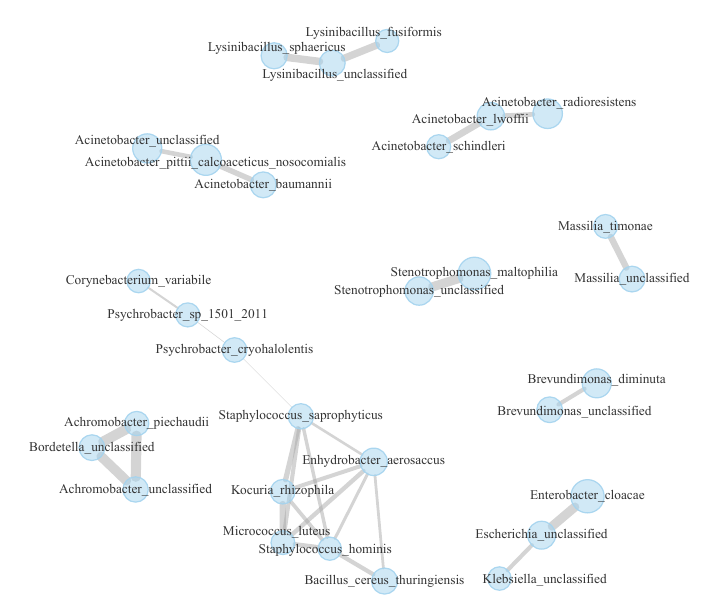


Supplementary Figure 3 - Co-occurrence clusters of microbial species for New-York City subway study data [(Afshinnekoo et al. 2015)](https://paperpile.com/c/Os3Gnk/oS5b).

**Supplementary tables**

Supplementary Table 1 - Sample information.

Supplementary Table 2 - List of pathogens detectable with the used method.

Supplementary Table 3 - Associations between microbial taxa and factors (n = 40 samples).
